# Advice Wanted

**Published:** 1893-02

**Authors:** W. E. Everly


### ADVICE WANTED.

Editor of The Homœopathic Physician:—Permit me, through the pages of your Journal, to ask the profession for a remedy to relieve the following group of symptoms:

Patient is thirty-five years old. Spare figure; irritable, fiery disposition. Easily discouraged; continually changing from one subject to another. Cannot complete one task before desiring to change to another one. Is never at rest. Always has something oppressing the mind. Cannot sit down either day or night to read or study without falling to sleep. When wakened feels very irritable and cross. Sleeps soundly at night. Rarely awakens before four or five o'clock in the morning. There are no other symptoms of importance, except a corn upon the right small toe, which burns and stings at times. When in the open air in cold weather a continual discharge from the nose of a watery, yellowish or green character.

It does not occur in a warm room.

W. E. Everly.

